# Vitamin D and omega-3 fatty acid supplements in children with autism spectrum disorder: a study protocol for a factorial randomised, double-blind, placebo-controlled trial

**DOI:** 10.1186/s13063-016-1428-8

**Published:** 2016-06-23

**Authors:** Hajar Mazahery, Cathryn Conlon, Kathryn L. Beck, Marlena C. Kruger, Welma Stonehouse, Carlos A. Camargo, Barbara J. Meyer, Bobby Tsang, Owen Mugridge, Pamela R. von Hurst

**Affiliations:** Institute of Food Science and Technology – School of Food and Nutrition, Massey University, Auckland, New Zealand; Commonwealth Scientific Industrial Research Organisation (CSIRO) Food, Nutrition and Bioproducts, Adelaide, SA Australia; Department of Emergency Medicine, Massachusetts General Hospital, Harvard Medical School, Boston, MA USA; School of Medicine, University of Wollongong, Illawarra, NSW 2522 Australia; North Shore Hospital, Waitemata District Health Board, Auckland, New Zealand

**Keywords:** Autism, ASD, Vitamin D, Omega-3 fatty acids, Supplements

## Abstract

**Background:**

There is strong mechanistic evidence to suggest that vitamin D and omega-3 long chain polyunsaturated fatty acids (n-3 LCPUFAs), specifically docosahexaenoic acid (DHA), have the potential to significantly improve the symptoms of autism spectrum disorder (ASD). However, there are no trials that have measured the effect of both vitamin D and n-3 LCPUFA supplementation on autism severity symptoms. The objective of this 2 × 2 factorial trial is to investigate the effect of vitamin D, n-3 LCPUFAs or a combination of both on core symptoms of ASD.

**Methods/design:**

Children with ASD living in New Zealand (*n* = 168 children) will be randomised to one of four treatments daily: vitamin D (2000 IU), n-3 LCPUFAs (722 mg DHA), vitamin D (2000 IU) + n-3 LCPUFAs (722 mg DHA) or placebo for 12 months. All researchers, participants and their caregivers will be blinded until the data analysis is completed, and randomisation of the active/placebo capsules and allocation will be fully concealed from all mentioned parties. The primary outcome measures are the change in social-communicative functioning, sensory processing issues and problem behaviours between baseline and 12 months. A secondary outcome measure is the effect on gastrointestinal symptoms. Baseline data will be used to assess and correct basic nutritional deficiencies prior to treatment allocation. For safety measures, serum 25-hydroxyvitamin D 25(OH)D and calcium will be monitored at baseline, 6 and 12 months, and weekly compliance and gastrointestinal symptom diaries will be completed by caregivers throughout the study period.

**Discussion:**

To our knowledge there are no randomised controlled trials assessing the effects of both vitamin D and DHA supplementation on core symptoms of ASD. If it is shown that either vitamin D, DHA or both are effective, the trial would reveal a non-invasive approach to managing ASD symptoms.

**Trial registration:**

Australian New Zealand Clinical Trial Registry, ACTRN12615000144516. Registered on 16 February 2015.

**Electronic supplementary material:**

The online version of this article (doi:10.1186/s13063-016-1428-8) contains supplementary material, which is available to authorized users.

## Background

Autism spectrum disorder (ASD) is a neurodevelopmental disorder usually diagnosed when developmental, educational and social demands increase [1]. ASD is believed to affect 1 % of the New Zealand population [1]. Diagnostic criteria for ASD include delays or difficulties in sociocommunicative functioning, restricted and repetitive behaviours/interests, sensory issues and aberrant behaviours [1, 2]. ASD is also associated with medical conditions such as gastrointestinal problems [1–5]. The clinical symptoms of individuals with ASD vary widely [5–7], suggesting that it is multi-factorial in nature.

It is generally agreed that both genetic and environmental factors contribute to the development of ASD. The high heritability of ASD has been shown by twin and familial studies [8, 9]. However, it has been reported that only 30 % of ASD cases are clearly associated with a syndrome or genetic markers leaving the aetiology of most cases without explanation [10].

Mechanistic evidence, as well as a scattering of ecological and cross-sectional studies, suggests that vitamin D may play an important role in the aetiology of ASD. Vitamin D receptors and 1α-hydroxylase have been identified in different regions of the brain and sensing neurons [11–13]. The active form of vitamin D has been shown to have an important role in the neuronal differentiation, structure, function and connectivity of the developing brain [14]. Vitamin D response elements have been identified on genes involved in serotonin and oxytocin synthesis [15]. Lower levels of plasma oxytocin [16] and abnormal serotonin concentrations in the brain and tissues outside the blood–brain barrier have been shown in populations with ASD [17, 18]. Oxytocin and serotonin have been implicated in modulating social behaviour [19, 20].

The serum level of 25-hydroxyvitamin D (25(OH)D), the best available marker of vitamin D status [21, 22], has been shown to be significantly lower in autistic individuals than in their healthy counterparts [23, 24]. Similarly, higher prevalence of ASD has been reported at higher latitudes and in individuals exposed to lower UVB radiation levels [24, 25]. In adults with severe autism living in a community centre in Italy, problem behaviours significantly increased during spring and decreased during autumn [26]. Depletion of vitamin D in body stores by the end of winter and early spring seasons (due to lack of sun exposure) may have exacerbated the symptoms of autism and increased problem behaviours observed in this study.

The potential role of vitamin D deficiency in autism has received surprisingly little attention. While a few case studies have reported beneficial effects of vitamin D supplementation on autistic core symptoms [27], no randomised, placebo-controlled trial with vitamin D supplementation has been conducted to date [28]. Jia et al. [27] reported that shifting serum 25(OH)D concentration in a child with ASD from 31 to 203 nmol/L after 2 months of high-dose vitamin D supplementation (150,000 international units (IU) per month administered intramuscularly plus 400 IU per day administered orally) improved autistic core symptoms. Although other trials investigating the effect of multivitamin/mineral supplements containing low doses of vitamin D on autism symptoms have provided promising results [29, 30], the individual effect of each nutrient cannot be determined from these studies.

Omega-3 long chain polyunsaturated fatty acids (n-3 LCPUFAs) also have the potential to positively affect children with ASD. These n-3 LCPUFAs, mainly DHA, are necessary for normal development and functioning of the brain and auditory and visual processing system [31–33]. Long-term DHA depletion results in significant losses in brain DHA with consequent loss of brain function [34]. Evidence shows that children with ASD have an increased omega-6 to omega-3 ratio in blood and low blood concentrations of n-3 LCPUFAs, which could be due to either low dietary intake or differences in fatty acid metabolism and incorporation into cellular membranes of children with ASD [35–37].

Reports on the benefits of n-3 LCPUFAs in treating ASD are inconclusive. There are, to our knowledge, only four randomised, placebo-controlled trials [38–41], three of which are small pilot studies. Bent et al. [40] and Amminger et al. [39] found that omega-3 supplementation was superior over a placebo (12 and 6 weeks, respectively) for reducing symptoms of hyperactivity and stereotypic behaviour in children with ASD. However, more recent studies have found that supplementation with n-3 LCPUFAs for 6 months had no beneficial effect on core symptom domains of ASD in children aged 2 to 5 years (*n* = 38) [41] and 3 to 10 years (*n* = 48) [38]. However, these studies are limited by their low participant numbers and short treatment periods.

In addition to these studies on the nutrients’ effects when given individually, there are speculations that vitamin D and n-3 LCPUFAs may improve ASD symptoms because of their shared functions and each nutrient-specific role that complements the other nutrient’s functions [42, 43]. Both nutrients are powerful anti-inflammatory agents, immune modulators and neuroprotectors [42]. Furthermore, evidence suggests that while vitamin D regulates serotonin synthesis, omega-3 fatty acids increase serotonin release and membrane fluidity and thus increase serotonin accessibility [43]. ASD is associated with increased inflammation, oxidative stress, immune dysregulation and/or mitochondrial dysfunction in brain regions that are involved in social behaviour, sensory and motor coordination, memory, speech and auditory processing, and also with neurotransmitter dysregulation [17, 18, 44].

Gastrointestinal problems have also been reported to be common in children with ASD. Compared to their typically developing siblings (12 %), autistic children have more gastrointestinal symptoms (42 %) [3–5]. Gastrointestinal problems may relate to abnormal gut flora [45], decreased activity of digestive enzymes [46] or increased intestinal permeability [47]. Vitamin D deficiency has been implicated in the pathophysiology of some gastrointestinal diseases, including inflammatory bowel disease [48], and thus might play a role in ASD-related gastrointestinal problems. Likewise, n-3 LCPUFAs have been shown to reduce symptoms of ulcerative colitis [49], to support epithelial integrity in vitro [50], and to alter the gut microbiota composition of both neurodevelopmentally normal and early life-stressed animals [51].

Unusual eating habits, a risk factor for nutrient deficiencies, are common in ASD [4]. Inadequate intakes of magnesium, zinc, folate, vitamins A, E, B_12_, K and D, as well as low intake of foods rich in n-3 LCPUFAs, have been reported in children with autism [52–58]. However, a broad picture of the nutritional status of affected children in New Zealand is lacking.

### Hypotheses

1. Both vitamin D and omega-3 status, defined as omega-3 index (red blood cell (RBC) DHA + eicosapentaenoic acid (EPA)), will be low in children with ASD at baseline (25(OH)D <75 nmol/L [59] and omega-3 index of approximately 4–6 % [60, 61])
2. Improving either vitamin D or omega-3 status with supplementation will reduce the severity of ASD symptoms in children with ASD
3. Combined vitamin D and n-3 LCPUFA supplementation will be more effective than either supplement alone or placebo in reducing the severity of ASD symptoms in children with ASD

### Aims

1. To establish the vitamin D and RBC fatty acid status of children with ASD living in Auckland, New Zealand
2. To investigate the effect of improving either vitamin D or omega-3 status in reducing the symptoms of ASD including sociocommunicative functioning, sensory issues and aberrant behaviours (primary outcomes) and gastrointestinal symptoms (secondary outcome)
3. To establish the effectiveness of supplementation with combined vitamin D and n-3 LCPUFAs in reducing the symptoms of ASD including sociocommunicative functioning, sensory issues and aberrant behaviours (primary outcomes) and gastrointestinal symptoms (secondary outcome)

## Methods/design

**Fig. 1:**
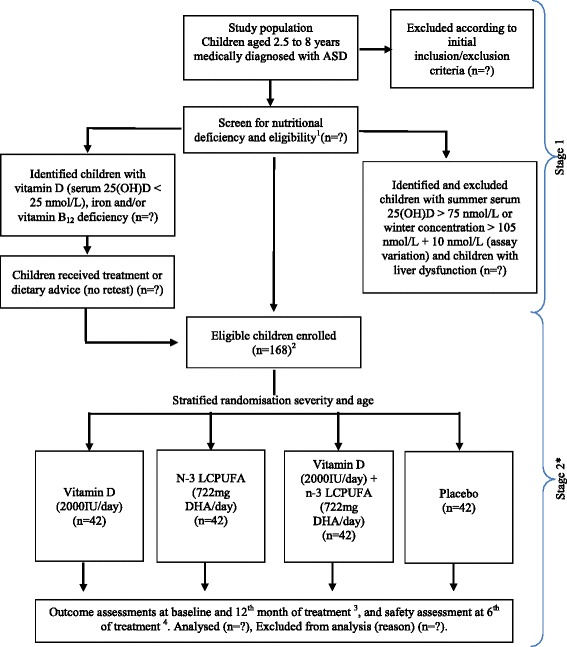
Schematic diagram of study design. ^1^Blood biomarkers: 25-hydroxyvitamin D (25(OH)D), red blood cell (RBC) fatty acids, calcium, albumin, iron studies, vitamin B_12_, folate, full blood count and vitamin A. Questionnaires: sociodemographics, medical history, eating/mealtime behaviours and food diary. ^2^Questionnaires: sociocommunicative functioning, sensory problems and aberrant behaviours (primary outcomes); gastrointestinal symptoms (secondary outcome), sun exposure and skin colour. Anthropometry: weight and height. ^3^Blood biomarkers: 25(OH)D, RBC fatty acids, calcium and albumin. Questionnaires: sociocommunicative functioning, sensory problems, aberrant behaviours (primary outcomes) and eating/mealtime behaviours and diet quality. Anthropometry: weight and height. ^4^Blood biomarkers: 25(OH)D, calcium and albumin. *Gastrointestinal symptoms (secondary outcome) and medication/supplement use/incidence of adverse events/supplement compliance will be monitored throughout the study period

This study consists of two stages: stage 1 will include recruitment and screening, while stage 2 is a vitamin D and n-3 LCPUFA randomised, double-blind, placebo-controlled trial (Fig. 1). The duration of the intervention is 12 months.

Stage 1 will provide the opportunity for a comprehensive description of the study population with respect to nutritional status (biochemical indices and dietary intake), demographics and medical history. Stage 2 will demonstrate the efficacy of supplementation with vitamin D, n-3 LCPUFAs or both on reducing ASD symptoms.

### Participants

This study is a collaboration between Massey University and the Waitemata District Health Board (WDHB), New Zealand. Caregivers of children who meet the criteria for the study will be approached in the first instance by the WDHB Developmental Coordinators.

We calculated that 42 participants (a minimum of 34 participants, and allowing for a 20-% potential dropout rate) would be required for each arm of the trial to demonstrate a clinically significant difference at 80 % power and 5 % statistical significance. Power calculations were based on a 17-unit difference between supplemented groups and placebo in change from baseline to endpoint on the Social Responsiveness Scale (SRS) total score [62], on a mean SRS and standard deviation of 105 and 24.7 units in untreated children with ASD, respectively (from our 2015 pilot study, unpublished). The sample size was calculated using the formula below [63]:

$$ N= 2{\alpha}^2K/{\left({\mu}_2\hbox{-} {\mu}_1\right)}^2 $$

where *N* is the sample size required per group, *α* is the SD, K is the constant (7.9 denotes 80 % power and 5 % significance), and (*μ*_2 −_*μ*_1_) is the difference in SRS total score between groups.

To ensure that the study is adequately powered, a blinded interim analysis at >50 % of the initially planned enrolment will be performed by an independent third party to estimate the variance for potential sample size increase.

### Inclusion and exclusion criteria

Children will be eligible for this study if they are aged between 2.5 and 8 years, have a medical diagnosis of ASD confirmed by both a developmental paediatrician in accordance with the criteria listed in the *Diagnostic and Statistical Manual of Mental Disorders, version five* (DSM-5) [2], and onset of symptoms after 18 months of age. The lower limit of 2.5 years has been chosen based on the age criteria of the psychological assessment tools, and the upper limit of 8 years has been chosen to avoid the confounding effect of behavioural changes associated with pubertal stage. The caregiver’s proficiency in English is a requirement (due to the nature of outcome assessment tools). Volunteers are excluded if they were diagnosed as having developmental delay since birth.

Additional inclusion criteria for the trial are: liver function within the normal range (albumin 34–48 g/L) and serum 25(OH)D <75 + 10 nmol/L if they enter the trial in winter and <105 nmol/L + 10 nmol/L if they enter the trial in summer. A 10-nmol/L variation was chosen because of the potential assay variability [64]. We have applied two different cut-off points for exclusion because there is a large seasonal variation in serum 25(OH)D concentrations in New Zealand ranging from 30 nmol/L [65] to 44 nmol/L [66].

### Setting

The study will take place in Auckland, New Zealand. Non-fasted blood samples will be collected at the North Shore or Waitakere Hospitals in Auckland, New Zealand. Questionnaires and anthropometry will be undertaken at Massey University, Auckland, New Zealand. Auckland is New Zealand’s largest city with a population of just over one million. It has been estimated that approximately 13,000 (32.5 %) individuals with ASD reside in the Greater Auckland region [1].

### Stage 1 – screening

Screening and recruitment (stage 1 of the study) will take place over a 24-month period, which commenced in January 2015. Children who meet the initial inclusion criteria will have a blood draw and will be screened for nutritional deficiencies. See Tables 1 and 2 for outcome measures, testing methods, and schedule of enrolment, intervention, and assessment, respectively.

**Table 1:** Summary of the study outcome measures and methods

Variables	Methods
Blood analysis^a^	
25(OH)D	Serum: Siemens ADVIA Centaur Vitamin D Total assay
RBC fatty acids	Erythrocytes: Shimadzu GC-17A flame-ionisation gas chromatography (Rydalmere, NSW, Australia)
Calcium	Serum: Siemens Dimension Vista® System. CA Flex® reagent cartridge, Cat. No. K1023
Albumin	Serum: Siemens Dimension Vista® System. ALB Flex® reagent cartridge, Cat. No. K1013
Iron studies	Ferritin, serum: Siemens Dimension Vista® System. FERR Flex® reagent cartridge, Cat. No. K6440
	Iron, serum: Siemens Dimension Vista® System. IRON Flex® reagent cartridge, Cat. No. K3085
	Total iron binding capacity, serum: Siemens Dimension Vista® System. TIBC Flex® reagent cartridge, Cat. No. K3084
Vitamin B_12_	Serum: Siemens Dimension Vista® System. B12 Flex® reagent cartridge, Cat. No. K6442
Folate	Serum: Siemens Dimension Vista® System. FOL Flex® reagent cartridge, Cat. No. K6444
Full blood count	Whole blood: Sysmex XE-5000™ Automated Haematology System
Vitamin A	Plasma:, Shimadzu Prominence Modular HPLC System. Cat. No. LC-20A. The assay was developed at Massey University
Questionnaires	
Primary outcome measures	
Sociocommunicative functioning	Social Responsiveness Scale-second edition (SRS-2) [73]. Completed by caregiver at Massey University
Sensory problems	Sensory Processing Measure (SPM) [75]. Completed by caregiver at Massey University
Problem behaviours	Aberrant Behaviour Checklist-Community (ABC-C) [78]. Completed by caregiver at Massey University
Secondary outcome measure	
Gastrointestinal symptoms	A questionnaire and diaries completed by caregiver
Other measures	
Sociodemographics	Completed by caregiver
Medical history	Completed by caregiver
Dietary assessments	Dietary Index Children’s Eating (DICE). Completed by caregiver
	Feeding issues and mealtime behaviours (Behavioural Paediatrics Feeding Assessment Scale, BPFAS). Completed by caregiver
	Four-day estimated food diary. Completed by caregiver. Analysed using FoodWorks 2007 (Xyris Software)
	Nutritional supplements and special dietary regimens. Completed by caregiver
Sun exposure and skin colour	Completed by caregiver
Medication/supplement use/incidence of adverse events and supplement compliance	Diary completed by caregiver
Anthropometry	Weight: Tanita electronic scale; Height: Stadiometre; measured by researcher at Massey University

*25(OH)D* 25-hydroxyvitamin D, *HPLC* high-performance liquid chromatography, *RBC* red blood cell

^a^All blood samples are collected and analysed at the North Shore and Waitakere Hospitals

**Table 2:** Schedule of enrolment, intervention, and assessment

	Study period						
	Enrolment		Allocation	Post allocation			
Timepoint	t_−2_	t_−1_	t_0_	t_baseline_	t_6-month_	t_12-month_	w_1–52_
Enrolment:							
Initial eligibility screen	♦						
Informed consent	♦						
Nutritional deficiencies screen		♦					
Allocation			♦				
Interventions:							
Vitamin D				♦	♦	♦	
Omega-3 LCPUFAs				♦	♦	♦	
Vitamin D + omega-3 LCPUFAs				♦	♦	♦	
Placebo				♦	♦	♦	
Assessments:							
Nutritional biomarkers							
Serum 25(OH)D				♦	♦	♦	
RBC fatty acids				♦		♦	
Calcium				♦	♦	♦	
Albumin				♦	♦	♦	
Iron studies				♦			
Vitamin B_12_				♦			
Folate				♦			
Full blood count				♦			
Vitamin A				♦			
Primary outcome							
Social Responsiveness Scale-2				♦		♦	
Sensory Processing Measure				♦		♦	
Aberrant Behaviour Checklist				♦		♦	
Secondary outcome							
Gastrointestinal symptoms				♦		♦	♦
Dietary assessment							
Dietary Index Children’s Eating				♦		♦	
Behavioural Paediatric Feeding Assessment Scale				♦		♦	
4-day food diary				♦			
Nutritional supplements and dietary regimen				♦			
Other assessments							
Sociodemographics				♦			
Medical history				♦			
Sun exposure and skin colour				♦			
Compliance and adverse events diary							♦

*25(OH)D* 25-hydroxyvitamin D, *t* timeline, *w* week, *LCPUFA* long chain polyunsaturated fatty acid

According to the SPIRIT statement: Defining Standard Protocol Items for Clinical Trials

### Pre-intervention preparation

Prior to randomisation and inclusion in the trial, vitamin D, iron and vitamin B_12_ deficiencies will be addressed. Refer to Table 3 for a list of nutritional deficiencies and the management strategies applied in this trial.

**Table 3:** Nutritional deficiencies and their management strategies prior to entering the intervention trial

Nutritional deficiency	Management
Vitamin D	Participants with serum 25(OH)D concentrations <25 nmol/L will be offered supplementation of 400 IU per day^a^
Iron	Children with iron deficiency will be offered iron supplements and postponed entry into the trial after 3 months. Children will not be retested A child will be iron deficient when two of the following pools are abnormal: red cell pool (haemoglobin <111 g/L, red blood cell distribution width >14 %), transport iron (iron saturation <16 %) and/or storage iron (serum ferritin ≤15 μg/L)^a^. Criteria for treatment will be according to the New Zealand Ministry of Health guidelines^b^
Vitamin B_12_	Children with serum levels <110 pmol/L will be offered the option of prescribed supplements or dietary advice to improve status

^a^New Zealand Ministry of Health 2015 [83]

^b^Retrieved from https://www.starship.org.nz/for-health-professionals/starship-clinical-guidelines/i/iron-deficiency/on 5 March 2015

### Stage 2 – vitamin D and n-3 LCPUFA intervention

The intervention consists of 2000 IU of vitamin D_3_ per day, 722 mg of DHA per day, 2000 IU of vitamin D_3_ plus 722 mg of DHA per day or placebo, in the form of four oral capsules. The treatment materials will be delivered in 750-mg gel capsules with a tear-off nozzle manufactured and supplied by Douglas Nutrition Ltd., Auckland, New Zealand. The study capsules, vitamin D, n-3 LCPUFAs and placebo are identical in appearance and all are tasteless and colourless. The child is required to consume the contents of four capsules per day mixed into their food of preference or by oral administration by syringe. Refer to Table 4 for the total daily intake and contents of each capsule. Throughout the study period, children are allowed to have any therapy or medication for autism as well as any supplement provided that it does not contain vitamin D or omega-3.

**Table 4:** Total daily intake of vitamin D and omega-3 long chain polyunsaturated fatty acids (n-3 LCPUFAs) and the contents of each capsule, vitamin D, n-3 LCPUFAs and placebo

Treatment groups	Daily intake
Vitamin D	2 × 750-mg capsules of olive oil, 2 × 1000-IU capsules of vitamin D_3_ in medium-chain triglycerides (MCT), alpha tocopherol
N-3 LCPUFAs	2 × 750-mg capsules of olive oil, 2 × high-DHA triglyceride fish oil capsules (total DHA dose = 722 mg/day), alpha tocopherol
Vitamin D and n-3 LCPUFAs	2 × 1000-IU capsules of vitamin D_3_ in 750 mg MCT, 2 × high-DHA triglyceride fish oil capsules (total DHA dose = 722 mg/day), alpha tocopherol
Placebo	750-mg capsules of olive oil plus alpha tocopherol (antioxidant)

*DHA* docosahexaenoic acid, *MCT* medium-chain triglycerides

Supplementation with 2000 IU vitamin D_3_ has been shown to be a safe dose in infants [67, 68], and the French Society of Paediatrics recommends 1000 to 1200 IU per day in breast-fed infants [69]. The body mass of 2.5–8 year-olds in our trial will be considerably greater than infants’, which should further reduce the risk of adverse events. Furthermore, 2000 IU per day is less than the safe upper limit of 2500 and 3000 IU/day suggested by the IOM for 1–3 and 4–8 year age groups, respectively [70].

The DHA dose of 722 mg/day is physiologically relevant and achievable through diet (equivalent to approximately three servings of fatty fish per week) and is comparable to doses used in other trials in children investigating the effects of n-3 LCPUFAs on behaviour and learning [71]. No side-effects have been reported in children with a dose of 600 mg DHA/day [72].

### Randomisation, blinding and concealed allocation

Children will be randomly allocated to one of four groups, having been stratified for age and severity of ASD. Randomisation of the active/placebo capsules, the randomised sequence list, and group assignment will be fully concealed from the researchers, children and caregivers for the entire study, including the data analysis. A third party not involved in any aspects of the study will generate a random block design in blocks of four and eight using Randomization.com (http://www.randomization.com/). The third party will allocate a treatment code to a child once their eligibility for the intervention is confirmed and the caregiver’s consent is received. In an emergency situation where breaking of the study blind will be required, plans will be in place for the principal investigator to contact the third party responsible for randomisation to reveal the treatment assignment for a given participant.

### Data collection

Participants (caregivers and children) will attend the Human Nutrition Research Unit (HNRU) at Massey University on two occasions: baseline and 12 months. Once recruited into stage 2 and prior to being given a 4-month supply of supplements, caregivers will complete some questionnaires on core symptoms of ASD (sociocommunicative functioning and sensory issues) and aberrant behaviours as the study primary outcome measures, and on gastrointestinal symptoms as the secondary outcome measure. Once the intervention is completed (12 months), baseline assessments of core symptoms of ASD and aberrant behaviours completed by caregivers will be repeated. Caregivers will also complete weekly gastrointestinal symptom diaries over the study period.

Further information will be collected to describe the study population characteristics at the baseline. This information includes eating/mealtime behaviours, food diaries, sun exposure, skin colour and anthropometry (weight and height). At the final visit, baseline assessment of eating/mealtime behaviours will be repeated and children’s weight/height will be measured. Refer to Table 1 for outcome measures and testing methods.

### Blood sampling and analysis

Children will have their blood samples drawn on three occasions, baseline (stage 1 – screening), 6 months (safety measures) and 12 months (endpoint). Refer to Table 1 for nutritional biomarkers and testing methods. The non-fasted blood samples will be collected under the supervision of paediatric staff and processed at North Shore or Waitakere Hospitals of WDHB laboratory service. Nutritional biomarkers will be assayed from a venous blood sample. These include the following: 25(OH)D, RBC fatty acids, calcium, albumin, iron studies, vitamin B_12_ and folate, full blood count and vitamin A. With the exception of RBC fatty acids and vitamin A, all biomarkers will be analysed at North Shore Hospital. RBC fatty acids will be analysed at the University of Wollongong, NSW, Australia, and vitamin A in a laboratory at Massey University, Auckland, New Zealand.

### Questionnaires

The primary outcome measures are psychological assessments of core symptoms of ASD and co-occurring problem behaviours which are detailed in Table 1. The secondary outcome measure is the assessment of gastrointestinal problems. Standardised instructions will be given to all caregivers on how to complete the questionnaires during their visit to the HNRU. Before the participant departs the HNRU researchers check all answers for completeness.

*Social Responsiveness Scale™, Second Edition (SRS-2)* [73]: the SRS-2 versions specific for age groups 2.5–4.5 and 4.5 through 18 years will be used. SRS-2 identifies social impairment associated with ASDs and quantifies its severity in the domains of social awareness, social information processing, reciprocal social communication, social anxiety/avoidance, and stereotypic behaviour/restricted interests [73]. The clinical validity and sensitivity of SRS-2 has been determined in populations with ASD [74].

*Sensory Processing Measures™(SPM)* [75]: the SPM versions specific for age groups 2–5 and 5–12 years will be used. SPM assesses sensory processing, planning and ideas (praxis) and social participation in children. The scales measure social participation, vision, hearing, touch, body awareness (proprioception), balance and motion (vestibular function) and planning and ideas (praxis) [75]. This tool has been standardised, validated and used in children with ASD [76, 77].

*Aberrant Behaviour Checklist (ABC)* [78]: the ABC measures the variety of behaviour problems, namely irritability, social withdrawal, stereotypic behaviour, hyperactivity and inappropriate speech [79]. It has been validated in children with ASD [80] and has been widely used in treatment outcome studies of ASD [40].

*Gastrointestinal symptoms questionnaire*: the gastrointestinal symptoms questionnaire is designed specifically for this study and includes gastrointestinal signs and symptoms most commonly reported in children with ASD [81, 82]. Caregivers of study participants will be provided with weekly online diaries and will be asked to record if the child had constipation, diarrhoea, flatulence, abdominal pain, vomiting/nausea, abdominal distension, unexplained daytime irritability or/and unexplained night-time awakening during the past week, and if so how many times these occurred. The gastrointestinal symptoms questionnaire has not been validated, but includes questions on gastrointestinal symptoms most widely reported in ASD populations, or behaviours that might be a consequence of gastrointestinal symptoms. We are not aware of a validated instrument for assessing gastrointestinal symptoms in autistic children, and certainly not in New Zealand.

*Dietary Index of Children’s Eating (DICE)*: the DICE is a simple diet-quality assessment tool which has been developed by the research team, based on the New Zealand Ministry of Health Food and Nutrition Guidelines for Healthy Children and Young People [83]. This tool has been validated in a cohort of New Zealand healthy children aged 2 to 8 years (unpublished), and will be validated in ASD children in this study against the 4-day estimated food diary and biochemical markers.

*Behavioural Paediatrics Feeding Assessment Scale (BPFAS)*: the BPFAS is a simple assessment tool which measures a child’s mealtime behaviour and parents’ attitudes and behaviours. It is a 35-item scale questionnaire comprising 25-likert scale (5 points) questions about child behaviour and 10 dichotomous questions about parents’ attitudes and behaviours. Cut-off scores for the BPFAS have recently been established [84]. The BPFAS is the most reliable parent-administered feeding questionnaire, with good internal validity and test-retest reliability [85].

*Four-day estimated food diary*: dietary intake data will be collected by a 4-day estimated food diary, including one weekend day. Instructions on how to accurately complete the food diary will be provided with the food diary. Participants will be given a free-post, pre-addressed envelope for the return of the booklet. Average macro and micronutrient intake will be assessed over four reported days using FoodWorks Professional Edition 7 (Xryis Software, Brisbane, QLD, Australia, 2012).

In the present study, a 4-day food diary was chosen because of the high respondent burden and time-consuming characteristics of longer food diaries such as a 7-day food diary. Because of the high within-person variation in nutrient intakes, it is recommended to record dietary intakes over a longer period of time to have a highly accurate estimate of intake. However, if a 4-day food diary covers different days randomly, it can provide accurate estimates of dietary intake [86].

Information regarding sun exposure and skin colour, nutritional supplements and any special dietary regimens followed will be collected by questionnaires specifically designed for the purpose of the current study. The sun exposure questionnaire includes a question on caregiver’s beliefs and attitudes toward sun exposure as well as questions on country and city of residency in pregnancy, season of birth and child’s sensitivity to temperature and light extremes.

### Compliance to medication/adherence to study protocol

Caregivers will receive weekly emails containing a link to the online compliance and gastrointestinal issues diary and a tip/fact about autism and nutritional matters. Caregivers will be contacted by telephone at 1, 3, 6 and 9 months for morale purposes and to encourage compliance. Caregivers also will receive quarterly trial newsletters. The newsletters will include an update on the study, generic topics about ASD, a caregiver’s experience in relation to that topic, and entertainments/competitions for the study children.

New intervention materials will be sent out to participants every 4 months and caregivers will be asked to place the bottles from the previous months aside, with any unused capsules in them, to be returned at their next visit (6 or 12 months) at which stage unused capsules will be counted and recorded. Compliance to treatment will be analysed by counting each participant’s remaining supplements once they have completed the intervention.

### Adverse events

All participants will be recalled at 6 months for a blood test to check for hypervitaminosis D (serum 25(OH)D >225 nmol/L) and hypercalcaemia (serum Ca >2.7 mmol/L). Results will be checked by a third party who is unblinded (but has no involvement in analysis of results). The third party will de-identify blood test results and send them to the trial paediatrician and senior investigator for review and recommendation if the child’s serum 25(OH)D or calcium level is above the safe upper limit. If 25(OH)D concentrations are at or approaching 225 nmol/L, or if hypercalcaemia is present, dose administration will be adjusted.

Compliance and gastrointestinal symptom diaries will be monitored on a weekly basis and all side effects will be recorded in the adverse events log to keep a track of the child’s general health and behavioural reactions. In the case of any adverse events, the child’s health will be monitored more closely for three to four consecutive weeks, and if the adverse event persists, the reports will be referred to the trial paediatrician and senior investigator for further investigation.

### Dissemination of results

Following the receipt and analysis of the food diary and the completion of the biochemical assays at screening, each participant will receive a feedback form. Anthropometric measurements and blood results (iron studies, vitamin B_12_, folate and full blood count) will also be included. Once recruitment into the trial is completed, participants who have not proceeded into the trial will receive notification of their vitamin D and RBC fatty acid levels.

On completion of the trial, participants will be informed of their baseline and end vitamin D and RBC fatty acid status, and whether they were taking the active or placebo dose. They will also receive a summary of psychological assessment outcomes of SRS-2 and SPM.

Participants and other stakeholders (such as health professionals, district health boards, primary health organisations, ASD support groups) will be given access to the study’s findings. Results will be presented at scientific conferences both nationally and internationally, prepared for publication in peer-reviewed journals, and circulated to the media.

### Data handling and statistical analysis

Name and address details will be maintained in Microsoft Excel. Check boxes will record the progress of a participant through the study. All other data will be entered into a single Microsoft Excel spreadsheet with participants identified only by their unique Subject Number. Scorings of the questionnaires will be double-checked by the psychologist and the researcher. All entries will be double-checked by another member of the research team. All documents will be stored safely under confidential conditions and archived for 5 years.

Statistical analysis will be performed using IBM SPSS version 21.0 (IBM Corp; released 2012. IBM SPSS Statistics for Windows Version 21.0. Armonk, NY, USA). Before commencement of statistical analysis the data will be cleaned and checked for coding errors. The data will be checked for plausibility by randomly checking the accuracy and completeness and verifying against source data. The variables will be tested for normality using the Kolmogorov-Smirnov test, the Shapiro-Wilk test and normality plots. Non-normally distributed data will be transformed into approximate normal distributions by logarithmic transformations. The data will be reported appropriately as mean (standard deviation) for normally distributed data; transformed data will be back-transformed from summary statistics into geometric mean (95 % CI), non-normally distributed data will be described as median (25th, 75th percentiles) and categorical data as frequencies.

Baseline characteristics of participants will be compared among groups using analysis of variance (ANOVA) for parametric data and the Kruskal-Wallis test for non-parametric data. The primary analysis, comparing the effects of treatment on symptoms of autism over 12 months, will be conducted using a generalised linear mixed-models procedure. Treatments and time will be included as fixed effects and the interactions between interventions and time will be tested. If significant main effects or interaction effects are observed, post-hoc analysis with Bonferroni adjustments will be performed. Potential confounding factors and effect modifiers (e.g. baseline 25(OH)D and RBC fatty acids, symptoms of autism at baseline, age and gender) will be investigated within the model. Logistic regression will be used to test the multiplicative interaction. Rothman’s synergy index, which would be equal to unity under additivity, and less than unity when suggesting antagonism, will be utilised to examine the postulated interaction effect of vitamin D and omega-3 LCPUFAs on core symptoms of ASD.

The secondary analysis, comparing the effects of treatment on gastrointestinal symptoms over 12 months, will be conducted using the same procedure. Potential confounding factors and effect modifiers (e.g. baseline diagnosis of any gastrointestinal symptoms and medication/supplement use) will be investigated within the model.

Differences between participants who complete and withdraw from the trial will be analysed using an independent *t* test or the Mann–Whitney test for continuous variables (e.g. age) and chi-square for categorical variables (e.g. gender).

Associations between severity of ASD symptoms and nutritional status (specifically, vitamin D, RBC fatty acids, iron and vitamin B_12_) at baseline will be assessed using regression analysis. The information will be used to assess treatment response according to tertiles of nutritional status showing significant relationship with severity of ASD.

Both intention-to-treat and per-protocol analyses will be utilised, though the primary method of analysis will be intention-to-treat. Statistical significance will be based on two-tailed tests with *P* <0.05 considered significant.

## Discussion

ASD is a life-long, disabling condition that is associated with deficits in social-communicative functioning, stereotypic behaviour and many behavioural and medical conditions including gastrointestinal symptoms [1, 2, 5–7]. The main purpose of this study is to measure the effect of vitamin D, n-3 LCPUFAs or a combination of both on the symptoms of ASD in affected children. There is a widespread interest in the mechanistic role of vitamin D and n-3 LCPUFAs in brain development and function, with some supportive clinical and epidemiologic studies. However, the effect of supplementing these nutrients on ASD pathogenesis and progression is not known. We anticipate that this trial will provide important insights into this causality of reported associations. As far as we are aware, no other randomised, double-blind, placebo-controlled trial has investigated the effects of vitamin D on symptoms of ASD, and the few trials that have been conducted with n-3 LCPUFAs [38–41] have been limited by small samples sizes and short trial duration and have shown conflicting results.

The strength of this project lies in its design: part 1 has been designed to provide insight into the nutritional status of children with ASD in New Zealand. Part 2 has been designed using a ‘Criterion Standard’ approach (randomised, double-blind, placebo-controlled trial) to investigate the effect of supplementation. The design minimises the effect of potential confounding factors by correcting some nutritional deficiencies prior to the trial entry and taking into account the effect of confounders and covariates on ASD symptoms over time. Our sample size and trial duration will also ensure an adequate power to detect clinically and statistically significant results.

If this trial is able to identify nutritional interventions that can make even a small difference to the lives of children with ASD by reducing their symptoms, the benefits will be considerable in terms of social and emotional well-being and educational achievements. Furthermore, it will also reduce the emotional, physical and financial strains among families or caregivers of autistic children and the wider societal networks. The potential benefits of the current study go beyond New Zealand and will affect all regions where ASD exists, and both vitamin D and omega-3 status below the optimal level is highly prevalent – the general trend of the most regions worldwide.

## Trial status

At the time of manuscript submission, the trial was recruiting participants.

## Abbreviations

25(OH)D, 25-hydroxyvitamin D; ABC, Aberrant Behaviour checklist; ASD, autism spectrum disorder; BPFAS, Behavioural Paediatrics Feeding Assessment Scale; CI, confidence interval; DHA, docosahexaenoic acid; DICE, Dietary Index of Children’s Eating; EPA, eicosapentaenoic acid; HNRU, Human Nutrition Research Unit; IU, international unit; n-3 LCPUFAs, omega-3 long chain polyunsaturated fatty acids; RBC, red blood cell; SPM, Sensory Processing Measures; SRS, Social Responsiveness Scale; WDHB, Waitemata District Health Board

## Additional files

Additional file 1: Information Sheet. (PDF 319 kb) Additional file 2: Consent Form-Main. (DOC 242 kb) Additional file 3: Consent Form-Storage of Blood Sample. (DOC 244 kb) Additional file 4: SPIRIT Checklist. (DOCX 50 kb)
