# Supplementary material for: Vitamin D and omega-3 fatty acid supplements in children with autism spectrum disorder: a study protocol for a factorial randomised, double-blind, placebo-controlled trial
Source: Trials. 2016 Jun 23;17:295. doi: 10.1186/s13063-016-1428-8 (PMC4917935; doi:10.1186/s13063-016-1428-8)
Supplement: Additional file 1: — Information Sheet. (PDF 319 kb) [file 13063_2016_1428_MOESM1_ESM.pdf]

MASSEY UNIVERSITY
COLLEGE OF HEALTH
TE KURA HAUORA TANGATA

The VIDOMA Study
A nutrition intervention in children with ASD
Information sheet for study participants

This information sheet provides you with the background to the research and other important details about what is involved, so please read carefully before deciding whether or not to participate.

We are currently recruiting children with Autism Spectrum Disorder (ASD) aged 2.5 - less than 8 years old to take part in this research. The study is nicknamed the VIDOMA study, short for **VI**tamin **D** and **OM**ega-3 in **A**utism. As a parent/guardian, it is important for you to understand why we doing this research and what it will involve for you if you decide that your child can participate. This information sheet tells you about the purpose of the study and what will happen to if you choose for your child to take part. Please take time to read it carefully and discuss it with others if you wish. Please ask if anything is not clear, or if you would like more information.

Introducing the researchers
This study involves a team of researchers from Massey University and from Waitemata District Health Board (WDHB).
The principal investigator is Dr Pamela von Hurst, Co-director of Massey's Vitamin D Research Centre. The study manager is Mr Owen Mugridge whose contact details are at the end of this information sheet. The psychologist on the team is Ms Lindy Thomas and the Paediatrician is Dr Bobby Tsang from WDHB. A number of other staff and students from the Division of Human Nutrition and Dietetics are also involved.

What is the purpose of this study?
Children with ASD often have deficiencies in their diet due to physical and behavioural issues related to the condition. Some of the nutrients which can be deficient in children with ASD are known to affect brain development and function. The two nutrients that this study is going to investigate are vitamin D and omega-3. Recent studies have shown that there are a variety of ways by which both these nutrients can affect the function and connectivity of the developing brain.

If shown to be effective, increasing the vitamin D and omega-3 status of children with ASD may be a powerful, non-invasive and low cost strategy for improving some of the symptoms of ASD and improving the quality of life for children and their families.

Why have I been chosen?
You have been invited to participate because your child has been diagnosed with Autism Spectrum Disorder.

Does my child have to take part?
No, it is completely up to you and your child to decide whether or not to take part. If you do decide to enter your child into the study you will be asked to sign a consent form. You are free to withdraw your child at any time without giving a reason. Your child will also be given a sheet describing what will happen (very simply) so that he or she has the opportunity to ask questions and agree to take part.

If you decide to withdraw your child from the study, data obtained to date may be kept and used to contribute to the overall results. However, if you request that your child's data and other information relating to your child are destroyed. Taking part in the study does not affect any aspect of the routine care your child received, or could receive from the local DHB.

What do I have to do?
You and your child will attend 5 appointments during the 12 month period. Your first visit will be at North Shore Hospital or Waitakere Hospital where your child will have a blood test. The blood test will look at vitamin D, iron, vitamin B12, folate, and magnesium. If your child is deficient in iron, vitamin B12 or magnesium, the study doctor will prescribe supplements to correct this, then your child can continue with the study. If your child has especially high vitamin D, he or she will not be able to continue with the study. However, we would like to continue with the collection of their dietary information which we will analyse and report back to you.

The second visit will be at Massey University, Albany. This visit will last for approximately 1.5 hours. Before you come, we will send you a “Preparation Kit” to prepare your child for all aspects of the study visit, to help familiarise them with the new surroundings and people. We will also send you a food diary to record 4 days of your child’s total food intake. At this visit you will be asked to complete some questionnaires about the symptoms of your child’s condition such as behaviour, sensory issues and socialisation. There will also be some questions regarding sun exposure habits of you and your child. We will measure the height and weight of your child and they will have time to play with some toys until you are finished.

As part of the study your child will have to take supplements. The supplements are in the form of a tasteless, colourless oil, and can be mixed in with your child’s food or drink, or administered with a medicine syringe. Your child will be randomised into a study group and you will be given the supplements that your child will take for the next 12 months. The supplements will either be vitamin D, Omega-3, a combination of vitamin D and Omega-3 or a placebo. The study is double-blinded meaning that neither you nor the researchers will know what your child is taking until the end of the study.

Six months after starting the study, another blood test will be required, at either North Shore Hospital or Waitakere Hospital. We will send you all the instructions for this at the time. Similarly, at the end of the 12 months there will be another blood test, and a visit to Massey University (and North Shore or Waitakere Hospital) for the same tests as were done at the beginning.

Altogether, you will make 5 trips to either the hospital and/or Massey University over the year of your child’s participation in the study. You are welcome to bring supporting friends or Whanau with you to any of the appointments.
Your child will have three blood tests during this period, and will have to take the supplement every day for 12 months.
There will also be questionnaires for you to complete at the beginning and end of the study.

What will happen to the blood samples?
Nearly all of the blood samples will be processed immediately at the North Shore Hospital Laboratory. A small sample of red cells will be saved and frozen for later analysis of red cell fatty acids. This analysis will be completed in a laboratory at the **University of Wollongong in Australia**. Some of the samples may not be analysed immediately after the study and will remain stored at the Massey Nutrition Laboratory in the meantime. These samples may be used for further analysis, including genetic analysis. There is a separate information sheet and consent form relating to these samples. If you are not comfortable with these aspects of the study, you do not have to consent to them. This will in no way affect your child’s participation in the rest of the study.

Will I be reimbursed for my time?
You will not receive reimbursement for your time. However, we will support you with travel for the trips made during the study, if requested.

What are the possible risks and disadvantages of taking part?
There are no reported risks of taking either vitamin D or Omega-3 at the dose we will be using. There is a very small risk that vitamin D supplementation will increase calcium levels in the blood. A safety check blood test will be carried out at the 6 month stage to ensure that all is going well with blood levels of vitamin D, Omega-3 and calcium.

It is quite possible that the blood test will cause your child (and you) some distress. The hospital staff are very experienced at conducting blood tests, and the Paediatric Registrar will be on hand if any further help is required. If you know that your child gets very distressed by blood tests, let us know and we can discuss with you the option of some light sedation which will be administered by the Paediatrician.

What are the advantages to taking part in the study?
As a participant in this study, your child will have a number of assessments which are not usually available through standard care from the District Health Board. Any nutritional deficiencies will be identified at the beginning of the study and addressed. You will receive a wealth of information about your child's responses to the psychological assessments which are normally valued at approximately $400. You will also be helping with research which, if successful, could make an important difference for many other children and families like yours.

What will happen if anything goes wrong?
The risks involved in this study are very small and all of the measurements are routinely made. If you have any concerns during the study you can discuss these with a member of the study team.

If your child has any other problems, illnesses or concerns you should discuss these with a member of the study team.

Any complaints you have will be fully investigated. If you have a concern about any aspect of this study, you should speak to a member of the study team who will do their best to answer your questions.

Will my taking part in the study be kept confidential?
Yes. All information collected about you and your child during the study will be kept strictly confidential. Information will be entered into a protected database at Massey University. Massey University code all data so that your names and address are kept separate from any other information about your child.

Information collected about you will be kept strictly confidential and secure in locked filing cabinets and/or electronic files on computers with passwords and restricted access. Each participant is assigned a unique code which is used on all data collected. Only the specified research team will have access to personal identifying information.

Massey University maintains a central record of all research projects but this does not include any personal information about participants. We will store the data for 10 years, at which point it will be destroyed.

What will happen to the results?
You will receive all the results that apply to your child. At the end of the study, we will also be in touch to let you know the results of the study. The overall results may be presented at scientific meetings or published in scientific journals to ensure that the wider community including health professionals know about the findings. The findings may also be featured in the media. Your child will not be identified in any of these presentations or publications.

Who is organising and funding the study?
This study is being co-ordinated by Massey University's College of Health in collaboration with Waitemata District Health Board. The Principal Investigator is Dr Pamela von Hurst. The study is funded from a number of sources including Douglas Nutrition Ltd who provided the supplements free of charge, and Massey University.

Who has reviewed the study?
This project has been reviewed and approved by the Health and Disability Ethics Committee: 14/NTA/113.

Contact for further information:
If you have any further questions or if you have any concerns whilst participating in the study then please contact Owen Mugridge – 09 213 6650.

Massey University, Albany
College of Health
Gate 4 – Building 80
Turitea Place
Albany 0632
Auckland
09 414 0800 Ext 43650

Compensation for Injury

If physical injury results from your participation in this study, you should visit a treatment provider to make a claim to ACC as soon as possible. ACC cover and entitlements are not automatic and your claim will be assessed by ACC in accordance with the Accident Compensation Act 2001. If your claim is accepted, ACC must inform you of your entitlements, and must help you access those entitlements. Entitlements may include, but not be limited to, treatment costs, travel costs for rehabilitation, loss of earnings, and/or lump sum for permanent impairment. Compensation for mental trauma may also be included, but only if this is incurred as a result of physical injury.

If your ACC claim is not accepted you should immediately contact the researcher. The researcher will initiate processes to ensure you receive compensation equivalent to that to which you would have been entitled had ACC accepted your claim.

Cultural Support

If you require Maori cultural support, talk to your whanau in the first instance. Alternatively you may contact the administrator for He Kamaka Waiora (Maori Health Team) by telephoning 09 486 8324 ext 2324

If you have any questions or complaints about the study you may contact the Auckland and Waitemata District Health Board's Maori Research Committee or Maori Research Advisor by telephoning 09 486 8920 ext 3204
