# Supplementary material for: Vitamin D and omega-3 fatty acid supplements in children with autism spectrum disorder: a study protocol for a factorial randomised, double-blind, placebo-controlled trial
Source: Trials. 2016 Jun 23;17:295. doi: 10.1186/s13063-016-1428-8 (PMC4917935; doi:10.1186/s13063-016-1428-8)
Supplement: Additional file 2: — Consent Form-Main. (DOC 242 kb) [file 13063_2016_1428_MOESM2_ESM.doc]

**The VIDOMA Study**

**PARTICIPANT CONSENT FORM**

I have read the Information Sheet and have had the details of the study explained to me. My questions have been answered to my satisfaction, and I understand that I may ask further questions at any time.

I agree for my child to participate in this study under the conditions set out in the Information Sheet.

| **Signature:** |  | **Date:** |  |
| --- | --- | --- | --- |
|  | | | |
| **Full Name of Parent/legal caregiver**  **Please print** |  | | |

| **Full Name of child**  **Please print** |  |
| --- | --- |
