# Supplementary material for: Vitamin D and omega-3 fatty acid supplements in children with autism spectrum disorder: a study protocol for a factorial randomised, double-blind, placebo-controlled trial
Source: Trials. 2016 Jun 23;17:295. doi: 10.1186/s13063-016-1428-8 (PMC4917935; doi:10.1186/s13063-016-1428-8)
Supplement: Additional file 3: — Consent Form-Storage of Blood Sample. (DOC 244 kb) [file 13063_2016_1428_MOESM3_ESM.doc]

**The VIDOMA Study**

**Storage of blood samples for future analysis**

**PARTICIPANT CONSENT FORM**

I have read the Information Sheet regarding the storage of blood samples for future analysis, including genetic analysis, and have had the details of the study explained to me. My questions have been answered to my satisfaction, and I understand that I may ask further questions at any time.

Please select one of the following:

- Yes, I agree to having part of my child’s blood sample held in storage for future analysis including genetic analysis under the conditions set out in the Information Sheet.
- No, please do not hold samples of my child’s blood for any future analysis, including genetic analysis.

| **Signature:** |  | **Date:** |  |
| --- | --- | --- | --- |
|  | | | |
| **Full Name of Parent Please print** |  | | |

|  |  |
| --- | --- |

| **Full Name of Child Please print** |  |
| --- | --- |
